# Supplementary material for: All-optical control of long-lived nuclear spins in rare-earth doped nanoparticles
Source: Nat Commun. 2018 May 29;9:2127. doi: 10.1038/s41467-018-04509-w (PMC5974411; doi:10.1038/s41467-018-04509-w)
Supplement: Supplementary file 1 — Supplementary Information [file 41467_2018_4509_MOESM1_ESM.pdf]

All-Optical control of long-lived spins in rare-earth nanoparticles

Serrano et al.

- Supplementary Information -

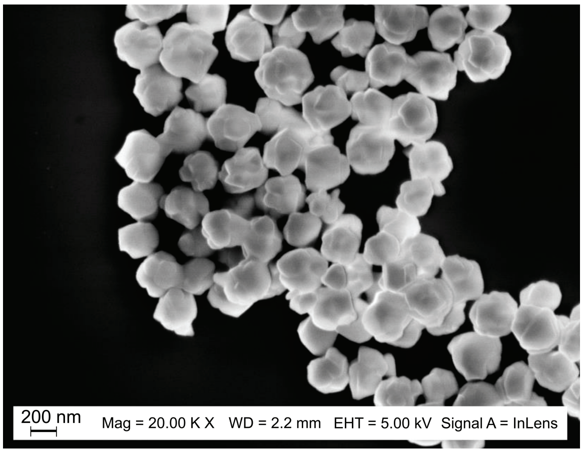

Supplementary Figure 1. Scanning electron microscopy (SEM) image of the Y_2O_3.:Eu^{3+} nanoparticles.

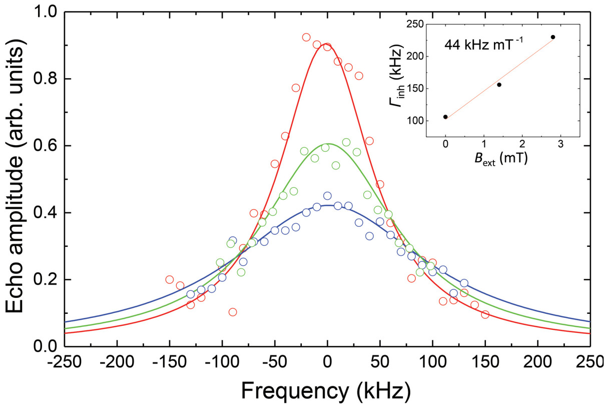

Supplementary Figure 2. Spin inhomogenous line broadening under magnetic field. $B_{\text{ext}} = 0$ (red), $B_{\text{ext}} = 1.4$ mT (green) and $B_{\text{ext}} = 2.8$ mT (blue). A broadening of the order of 44 kHz mT^{-1} is observed which is attributed to the random orientation of the particles regarding the external field. Spin dynamics investigations are therefore very challenging at higher fields due to the reduced peak absorption resulting from broadening.

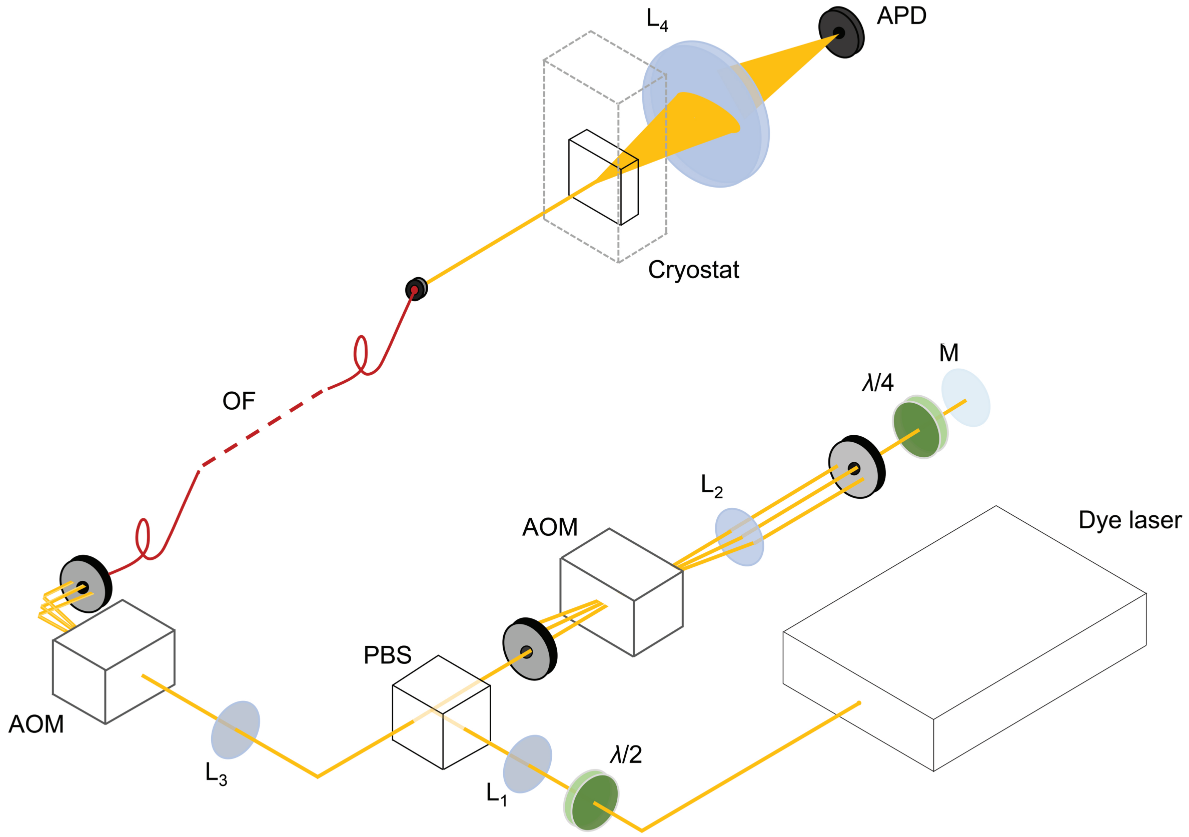

Supplementary Figure 3. Experimental setup. AOM stands for acousto-optic modulator, PBS for polarising beam splitter, OF for optical fibre, APD for avalanche photo diode and L_n represent the different lenses in the setup. Unlabelled elements correspond to pinholes.

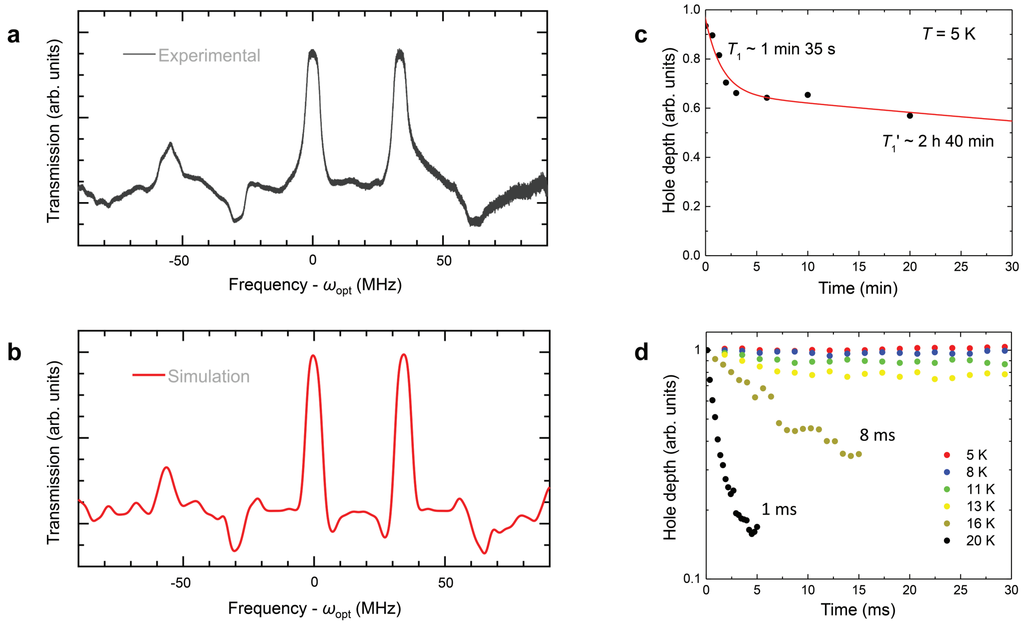

Supplementary Figure 4. Spin initialisation and spectral hole dynamics. **a.** Experimental and **b.** simulated optical preparation with $\omega_{\rm opt}$ corresponding to the optical transition frequency: 516.098 THz (580.883 nm vac.). **c.** Spectral hole dynamics at 5 K. **d.** Spectral hole dynamics as a function of temperature showing clear T_1 shortening for $T > 16$ K.

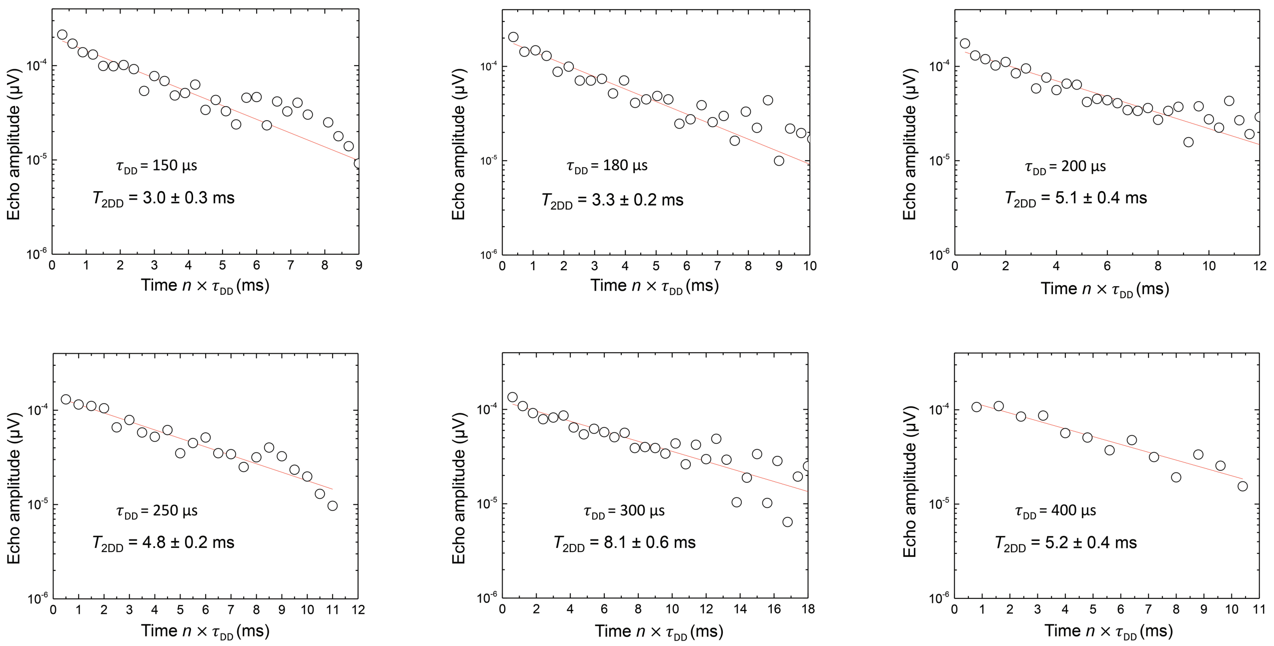

Supplementary Figure 5. Dynamical decoupling decays for different pulse separations τ_{DD}. $T_{2\mathrm{DD}}$ values are obtained by single-exponential fit.

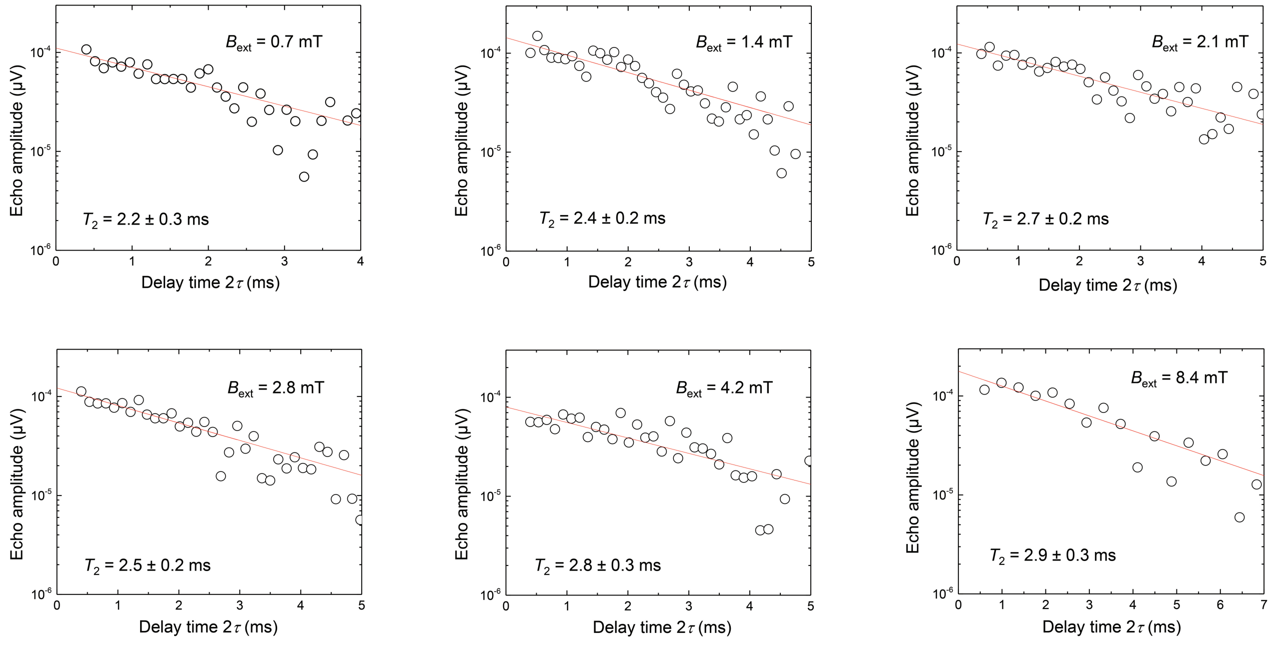

Supplementary Figure 6. Spin echo decays and coherence lifetime evolution for different external magnetic fields. T_2 values are obtained by single-exponential fit.

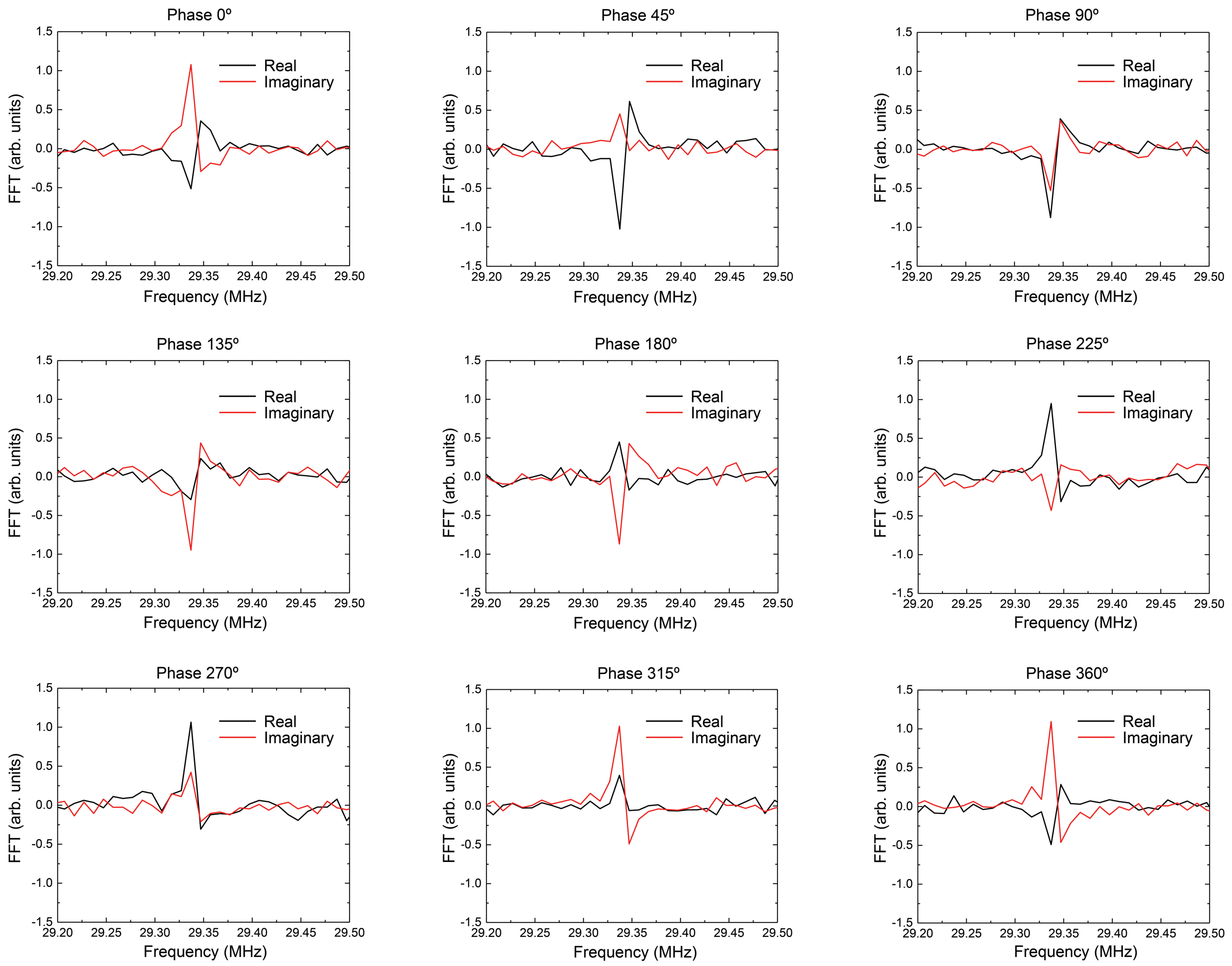

Supplementary Figure 7. Real and Imaginary parts of the heterodyne signal FFT for different excitation phases in the two-pulse echo measurements, with a delay time τ equal to 300 μs.

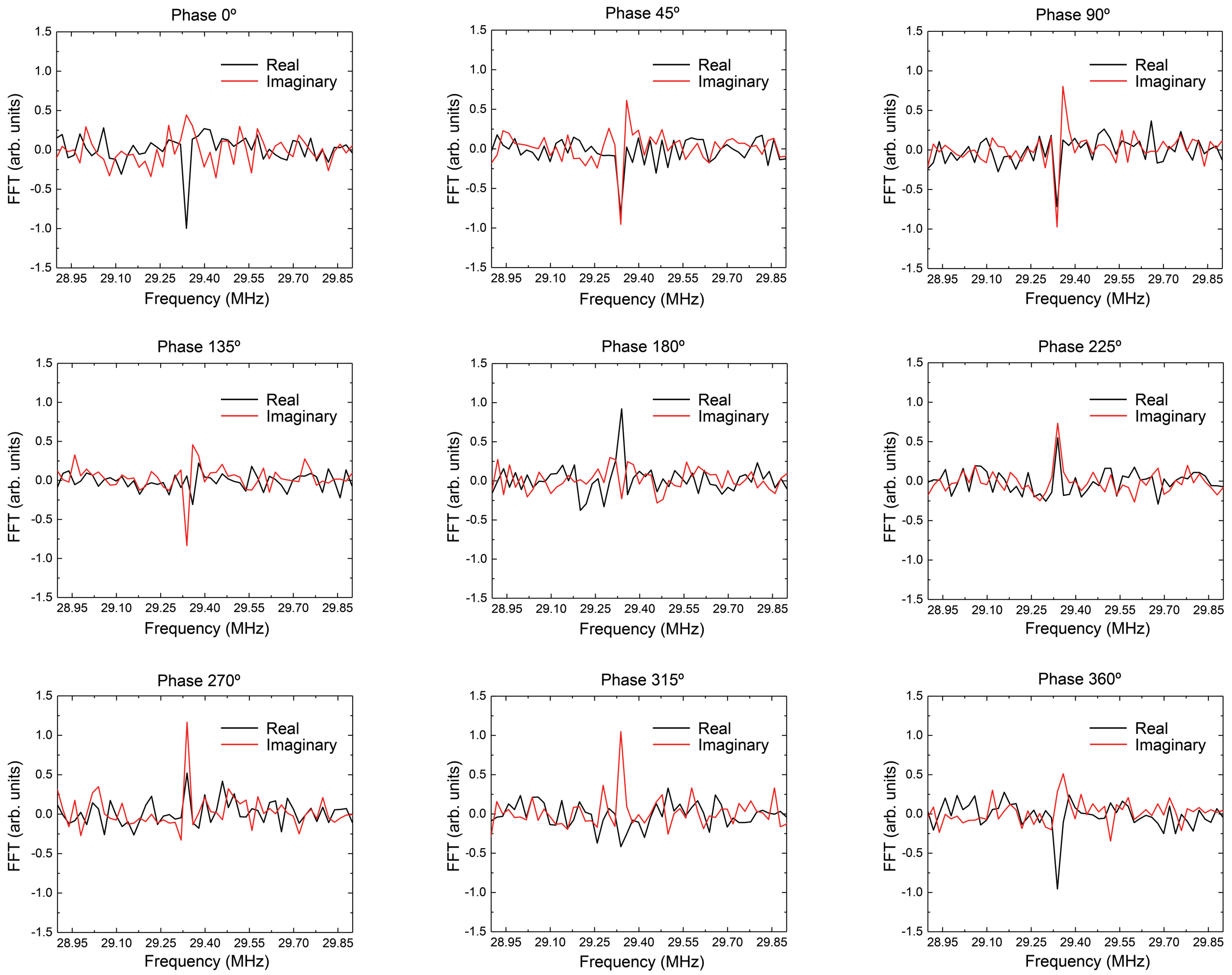

Supplementary Figure 8. Real and Imaginary parts of the heterodyne signal FFT for different excitation phases after 10 π pulses in a DD sequence, with a pulse separation τ_{DD} equal to 150 μs.

Supplementary Discussion

Optical preparation and hyperfine relaxation

The experimental transmission spectrum obtained after burning two spectral holes at 0 and 33.99 MHz, resulting from the application of optical pulses at frequencies referred to as ω_2 and ω_3 in the main manuscript (Fig. 1), can be simulated assuming equal branching ratios between excited and ground state hyperfine levels, and negligible spin relaxation rates. Both 151 and 153 europium isotopes have been taken into account in the simulation. The good agreement between the experimental and simulated spectra is evidenced in Suppl. Figs. 4a and 4b. The efficiency of the optical preparation given as the percentage of induced transparency was determined by comparing the prepared transmission spectrum at the centre of the optical absorption line (580.883 nm) with the off-line transmission (full transmission).

The analysis of the spectral hole dynamics enables us to estimate the hyperfine population lifetime (T_1) and to confirm that T_2 is here not fundamentally limited by population relaxation. The measurement was carried out by burning a spectral hole and progressively increasing the readout time. As displayed in Suppl. Fig. 4c, two decay regimes can be observed. This indicates the existence of different spin-lattice relaxation rates in the particles, of the order of 4.2×10^{-2} s^{-1} and 1×10^{-4} s^{-1} respectively. Nevertheless, even the fastest relaxation rate is still orders of magnitude smaller than the spin relaxation rate of 7.7×10^{2} s^{-1} corresponding to a coherence lifetime T_2= 1.3 ms at zero field (Fig. 2b). T_2 is therefore far from being limited by T_1 at 5 K (Suppl. Fig. 4d).

Dynamical decoupling (DD) modelling

A model based on frequency fluctuations following a Gaussian process[1] gives the relation between coherence lifetime $T_{2\mathrm{DD}}$ and the delay time between π pulses, τ_{DD}, as:

$$\frac{1}{T_{2\mathrm{DD}}} = \sigma_\Delta^2\tau_\mathrm{c}(1 - \frac{2\tau_\mathrm{c}}{\tau_{\mathrm{DD}}}\tanh(\frac{\tau_{\mathrm{DD}}}{2\tau_\mathrm{c}})) \tag{1}$$

in which τ_c is the correlation time and σ_Δ the standard deviation of the fluctuations. For $\tau_\mathrm{c} << \tau_{\mathrm{DD}}$ single exponential decays are expected with decay rate:

$$\frac{1}{T_{2\mathrm{DD}}} = \sigma_\Delta^2\tau_\mathrm{c} \tag{2}$$

Using Eqs. 1 and 2, σ_Δ and τ_c were determined by a best fit to $T_2 = 1.3$ ms (Fig. 2b, $B_{ext} = 0$), T_{2DD} ($\tau_{DD} = 300$ μs) = 8.1 ms and T_{2DD} ($\tau_{DD} = 400$ μs) = 5.2 ms (Suppl. Fig. 5). The values obtained were σ_Δ=2π×320 Hz and $\tau_c = 0.2$ ms. Nevertheless, this first analysis assumes pulse area errors to be negligible. In order to estimate their influence on σ_Δ and τ_c, we added an extra contribution to the dephasing as follows: applying a pulse of area $\pi - \alpha$, where α is considered small, reduces a given coherence ρ by a factor $|1 - \alpha^2/2|$. Thus, after N pulses, ρ reads:

$$|\rho(N)| = \left|\rho(0)(1 - \alpha^2/2)^N\right| \tag{3}$$

or after a time $t = N\tau_{DD}$:

$$|\rho(N)| = \left|\rho(0)(1 - \alpha^2/2)^{\frac{t}{\tau_{DD}}}\right| \tag{4}$$

This results in an exponential decay with a rate:

$$W = \frac{-\ln(1 - \alpha^2/2)}{\tau_{DD}} \tag{5}$$

However, in our experiments W increases faster than ${\tau_{DD}}^{-1}$ for $\tau_{DD} < 300$ μs and we modelled it by a phenomenological expression:

$$W = \frac{a}{(\tau_{DD}/\tau_0)^b} \tag{6}$$

A fit to the experimental data is shown in the manuscript in Fig. 3d. The fitted values for σ_Δ=2π×300 Hz and $\tau_c = 0.210$ ms are only slightly different than those found when assuming no pulse errors. The other parametres are $b = 4.9$, and $a = 4900$ s^{-1}, with τ_0 fixed at 100 μs.

Analysis of the optical and spin transition sensitivity to magnetic and electric perturbations

The spin coherence results reported in this research work evidence spin coherence is more preserved than optical coherence at the nanoscale[2]. Optical coherence is therefore concluded to be limited by mechanisms which are different to those limiting the spin coherence in the nanoparticles, related to the distinct sensitivity of optical and spin transitions to electric and magnetic perturbations.

The $^5D_0$ and $^7F_0$ levels are well separated from other electronic levels (ΔE ($^5D_0$-$^5D_1$) = 1600 cm^{-1} and ΔE ($^7F_0$-$^7F_1$) = 170 cm^{-1}), which reduces the second order electronic contribution to the hyperfine gyromagnetic factor. In the ground state, average gyromagnetic factors of 2.8 and 7.1 MHz T^{-1} have been measured for $^{151}EuCl_3.6H_2O$[3] and $^{151}Eu^{3+}$:Y_2SiO_5 respectively[4]. On the other hand, the quadratic Zeeman contribution to the optical transition is also negligible for small magnetic fields[3]. We therefore expect the optical transitions to have magnetic sensitivity similar to the ground state spin ones within a factor of about 5 at most. In contrast, the sensitivity of the optical and spin transition to electric noise in concluded to be remarkably different. An average Stark coefficient of 50 kHz V^{-1} cm^{-1} has been determined for the optical transition in Eu^{3+}:Y_2O_3 ceramics. The spin Stark coefficient is however much lower, on the order of 1 Hz V^{-1} cm^{-1})[5], in other words, spin transitions are basically insensitive to electric noise due to the weak coupling of nuclear spins to electric fields. The large difference between the optical and spin Stark coefficients means that an electrical noise causing a shift of 10 Hz on the spin will result in a 500 kHz shift on the optical transition. This is in agreement with the hypothesis that electric noise, which can be due to surface charge fluctuations or charged defects ionisation mechanisms in the nanoparticles[2], is the major limitation to optical coherence. In contrast, magnetic perturbations are concluded to remain the major cause for spin dephasing.

T_2 evolution with external applied magnetic field

The application of a external magnetic field provides a further insight into the dephasing mechanism limiting spin T_2. The observed single exponential character of the spin echo decays under external magnetic field (Suppl. Fig. 6) and the correlation time $\tau_c \sim$ 0.2 ms derived from the dynamical decoupling results[1], point to a decoherence source which fluctuates faster than the Eu^{3+} spin evolution time. Furthermore, the resulting

relaxation rate ${\tau_c}^{-1} = 5$ kHz, seems to confirm that, in contrast to bulk crystals[6] and ceramics[7], the magnetic centers responsible for the frequency perturbations are not ^{89}Y nuclear spins since their relaxation rate is expected to be very slow, typically of the order of 10 Hz according to results reported in bulk crystals[5]. A two-order of magnitude increase would therefore be needed in the nanoparticles to account for our results. Moreover, ^{89}Y spins relaxation is dominated by flip-flop processes which are very weakly dependent on the magnetic field. Thus, it seems more likely that magnetic defects carrying electron spins are responsible for the observed Eu^{3+} spin dephasing in the nanoparticles. Such defects, like electrons trapped in oxygen vacancies, have been identified in transparent Eu^{3+}:Y_2O_3 transparent ceramics, where they are thought to contribute to dephasing on the optical transitions as shown in reference[8].

The possibility of a direct effect of the external magnetic field (B_{ext}) on the spin bath dynamics, in particular, an acceleration of the environmental spin relaxation rate R (with $R \propto \alpha_{\mathrm{D}} B^5$ and α_{D} the phonon coupling constant[8]) was first considered as the mechanism behind the homogenous line narrowing observed in Fig. 2c. Nevertheless, the applied fields (few mT) are too weak to account for the observed behaviour since α_{D} values as high as 70 kHz mT^{-5} were derived by fit to be required to reproduce the observed experimental results. Such value is many orders of magnitude larger than standard α_{D} values reported for electronic spins[9,10], varying from Hz T^{-5} to a maximum of kHz T^{-5}. Instead, the observed experimental behavior was found to be well described by the evolution of the magnetic dipole-dipole (dd) interaction ($\hat{H}_{\mathrm{dd}}$) between one Eu^{3+} ion and environmental spins, here modelled as defects with electron spin S=1/2 and g-factor of 2. As reported in[11], weak magnetic fields can reduce the dd interaction Hamiltonian to secular terms decreasing the Eu^{3+} nuclear spin frequency shifts due to spin flipping in the surrounding spin bath.

For Eu^{3+}-defect pairs we consider the total Hamilonian of the system as:

$$\hat{H}_{\mathrm{tot}} = \hat{H}_{\mathrm{ZEu}} + \hat{H}_{\mathrm{Zdef}} + \hat{H}_{\mathrm{dd}} \tag{7}$$

with $\hat{H}_{\mathrm{ZEu}}$ and $\hat{H}_{\mathrm{Zdef}}$ the respective nuclear and electron Zeeman terms for Eu^{3+} and electron spin defects which were calculated as:

$$\hat{H}_{\mathrm{ZEu}} = -\gamma_{\mathrm{Eu}} \mathbf{B} \cdot \mathbf{I} \tag{8}$$

$$\hat{H}_{\mathrm{Zdef}} = \mu_{\mathrm{B}} g_{\mathrm{def}} \mathbf{B} \cdot \mathbf{S} \tag{9}$$

in which S=1/2, g_{def}=2, I_{Eu}=5/2 and γ_{Eu}=5 MHz T^{-1}. $\hat{H}_{\mathrm{dd}}$ is the dd interaction term expressed as:

$$\hat{H}_{\mathrm{dd}} = \frac{\mu_0}{4\pi} \frac{\gamma_{\mathrm{Eu}} \mu_{\mathrm{B}} g_{\mathrm{def}}}{r^3} (3(\mathbf{S} \cdot \mathbf{n})(\mathbf{I} \cdot \mathbf{n}) - \mathbf{S} \cdot \mathbf{I}) \tag{10}$$

where μ_0 is the vacuum permeability, r is the Eu^{3+}-defect distance and $\mathbf{n}$ the unit vector joining them. The eigenvalues of the $\hat{H}_{\mathrm{dd}}$ term were numerically determined for increasing magnetic fields as the difference between the eigenvalues of the total Hamiltonian $\hat{H}_{\mathrm{tot}}$ and those of the total Zeeman Hamiltonian $\hat{H}_{\mathrm{Z}}$=$\hat{H}_{\mathrm{ZEu}}$+$\hat{H}_{\mathrm{Zdef}}$ averaging over distances r from 0.22 nm (Y-O shortest distance) and relative orientations. The evolution of the obtained $\hat{H}_{\mathrm{dd}}$ eigenvalues with B_{ext}, having a direct impact in σ_{Δ} (Eq. 2), results in T_2 extension in excellent agreement with the experimental results in Fig. 2c. Furthermore, the calculation, carried out for a single Eu^{3+} ion, allows to estimate the concentration of defects by normalisation to the experimental values. The defect concentration is therefore estimated to c=6.4×10^{17} cm^{-3}, approximately equivalent to 25 ppm. This concentration of defects is 200 times lower than the Eu^{3+} concentration (5000 ppm). Thus, there are 2×10^4 defects per 4×10^6 Eu^{3+} ions in a single particle.

Supplementary References

[1] Pascual-Winter, M. F., Tongning, R. C., Chanelière, T. & Le Gouët, J. L.,Spin coherence lifetime extension in Tm^{3+}:YAG through dynamical decoupling, *Phys. Rev. B* **86**, 184301 (2012).

[2] Bartholomew, J. G., Oliveira Lima, K., Ferrier, A. & Goldner, P., Optical Line Width Broadening Mechanisms at the 10 kHz Level in Eu^{3+}:$\mathrm{Y_2O_3}$ Nanoparticles, *Nano Lett.* **17** (2), 778 (2017).

[3] Ahlefeldt, R. L., Zhong, M., Bartholomew & J. G., Sellars, M. J., Minimizing Zeeman sensitivity on optical and hyperfine transitions in $\mathrm{EuCl_3 \cdot 6H_2O}$ to extend coherence times, *J. Lum.* **143**, 193 (2013).

[4] Longdell, J., Alexander, A., L. & Sellars, M. J., Characterization of the hyperfine interaction in europium-doped yttrium orthosilicate and europium chloride hexahydrate, *Phys. Rev. B* **74** 19, 195101 (2006).

[5] Macfarlane, R. M., Arcangeli, A., Ferrier & A., Goldner, P., Optical measurement of the effect of electric fields on the nuclear spin coherence of rare-earth ions in solids, *Phys. Rev. Lett.* **113** 15, 157603 (2014).

[6] Arcangeli, A., Lovric, M., Tumino, B., Ferrier, A. & Goldner, P., Spectroscopy and coherence lifetime extension of hyperfine transitions in $^{151}Eu^{3+}:Y_2SiO_5$, *Phys. Rev. B* **89**, 184305 (2014).

[7] Karlsson, J., Kunkel, N., Ikesue, A., Ferrier, A. & Goldner, P., "Nuclear spin coherence properties of $^{151}Eu^{3+}$ and $^{153}Eu^{3+}$ in a Y_2O_3 transparent ceramic", J. Phys: Condens. Matter **29**, 125501 (2017).

[8] Kunkel, N., Barthomomew, J., Binet, L., Ikesue and A., Goldner, P., High-Resolution Optical Line Width Measurements as a Material Characterization Tool, *J. Phys. Chem. C*, **120** (25), 13725 (2016).

[9] Böttger, Thiel, C., Sun, Y., Cone, R. L., Optical decoherence and spectral diffusion at 1.5 μm in $Er^{3+}:Y_2SiO_5$ versus magnetic field, temperature, and Er^{3+} concentration, *Phys. Rev. B* **73**, 075101 (2006).

[10] Lutz, T. et al, Effects of mechanical processing and annealing on optical coherence properties of $Er^{3+}:LiNbO_3$ powders, *J. Lum.* **191**, 2 (2017).

[11] DeVoe R., G., Wokaun, A., Rand, S. C. & Brewer, R. G., Monte Carlo theory of optical dephasing in $LaF_3:Pr^{3+}$, *Phys. Rev. B* **23** (7), 3125 (1981).
